# Germline Mutations in Patients With Early-Onset Prostate Cancer

**DOI:** 10.3389/fonc.2022.826778

**Published:** 2022-06-06

**Authors:** Tang Tang, Xintao Tan, Ze Wang, Shuo Wang, Yapeng Wang, Jing Xu, Xiajie Wei, Dianzheng Zhang, Qiuli Liu, Jun Jiang

**Affiliations:** ^1^ Department of Urology, Daping Hospital, Army Medical University, Chongqing, China; ^2^ Genetron Health (Beijing) Co., Beijing, China; ^3^ Department of Bio-Medical Sciences, Philadelphia College of Osteopathic Medicine, Philadelphia, PA, United States

**Keywords:** prostate cancer, early-onset, next-generation sequencing, germline mutations, homologous recombination associated genes

## Abstract

**Objective:**

To investigate the inherited mutations and their association with clinical features and treatment response in young-onset prostate cancer patients.

**Method:**

Targeted gene sequencing on 139 tumor susceptibility genes was conducted with a total of 24 patients diagnosed with PCa under the age of 63 years old. Meanwhile, the related clinical information of those patients is collected and analyzed.

**Results:**

Sixty-two germline mutations in 45 genes were verified in 22 patients. *BRCA2* (20.8%) and *GJB2* (20.8%) were found to be the most frequently mutated, followed by *CHEK2, BRCA1, PALB2, CDKN2A, HOXB13, PPM1D*, and *RECQL* (8.3% of each, 2/24). Of note, 58.3% (14/24) patients carry germline mutations in DNA repair genes (DRGs). Four families with HRR (homologous recombination repair)-related gene mutations were described and analyzed in detail. Two patients with BRCA2 mutation responded well to the combined treatment of androgen deprivation therapy (ADT) and radiotherapy/chemotherapy.

**Conclusion:**

Mutations in DRGs are more prevalent in early-onset PCa with advanced clinical stages, and these patients had shorter progression-free survival. ADT Combined with either radiotherapy or chemotherapy may be effective in treating PCa caused by HRR-related gene mutations.

## Introduction

Prostate cancer (PCa) is the second most common cancer in men worldwide (1). More recent data indicate that genetic mutations predispose the patients to PCa (2, 3), evidenced by more than 60 loci linked to approximately 30% of familial PCa (4). It has been estimated that about 42% PCa risks, especially for the early-onset PCa, are attributable to genetic alterations (5). Mutations in DRGs especially HRR-related genes such as *BRCA1* and *BRCA2* are the well-established risk factors for PCa (6), with approximately 10% PCa patients harboring deleterious mutations in DRGs (7). Furthermore, compared with those carrying somatic mutations in DRGs, patients with hereditary germline mutations have an earlier onset, a higher propensity to metastasis, and shorter progression-free survival (8).

HRR is the most efficient repair tool for DNA double-strand breaks (DSB) with high fidelity (9). BRCA1/2, together with their functional-related factors such as ATM, ATR, and PALB2, form HRR complexes and play pivotal roles in maintaining genome stability (9). Cells with mutations in any of these genes are more vulnerable to DNA damage and cancer development, especially when they are exposed to either endogenous or exogenous attacks (10, 11). Therefore, mutations in HRR-related genes have been frequently observed in different cancer types, including breast, ovarian, pancreatic, stomach, laryngeal, and PCa (12). The *BRCA1/2* mutations are predominantly observed in breast and ovarian cancer in women (13) and PCa in men (14). Germline *BRCA1/2* mutations are also associated with higher Gleason scores (≥8), a more aggressive phenotype with a higher probability of nodal involvement, distant metastasis, and shorter overall survival (15, 16). Germline *BRCA2* and *BRCA1* mutations present in 1.2% and 0.44% PCa (17), respectively. The frequency of *BRCA2* mutation in early-onset PCa patients (<65 years old) could be as high as 2.2% (18). Based on these data, we hypothesized that germline mutations could be one of the important underlying mechanisms for PCa early onset. To test this hypothesis, we investigated the frequency of genetic mutations of 139 tumor-susceptibility genes in 24 young-onset PCa patients. We found that mutations in DRGs are not only more prevalent in early-onset PCa, patients with these mutations also have much shorter progression-free survivals.

## Patients And Methods

### Patients

Initially, 239 PCa patients admitted to Daping hospital between September 2016 and December 2020 were screened for this study with the protocol approved by the ethics committee of the Third Affiliated Hospital of the PLA Army Medical University (ethics number: 2018 No. 28). The following criteria were used for inclusion:

1) Patients that had been pathologically diagnosed with prostate cancer
2) Age at diagnosis is younger than 65
3) Informed consent: volunteered to participate in this study
Most individuals were excluded due to the following criteria:
1) Limited sample availability or unqualified samples
2) Unwillingness to participate in this study
3) Unavailability of following-up data

A total of 24 PCa patients were included in the study with their clinical data, including ages, PSA levels at diagnosis, biopsy Gleason score, pathological types from needle biopsy and surgical specimens, metastasis status, clinical stages, androgen deprivation therapy (ADT) strategies, and gene sequencing results. Additionally, the levels of testosterone and PSA during ADT were monitored. The diagnosis of castration-resistant prostate cancer was made during follow-up based on the American AUA guidelines (2018 edition): (1) Serum testosterone <50ng/dL (<1.7nmol/L); (2) Elevated PSA: a PSA increase of >2ng/ml from the lowest level at a minimum of 3 weeks interval or an increase of 25% from the lowest level at the second measurement. Two or more new lesions detected in a bone scan are indicative of disease progression as well.

### Sample Collection and gDNA Library

Peripheral blood was collected from the elbow vein into EDTA-containing tubes and stored at 4°C for further examination. Total DNA is extracted from the mononuclear cells. AllPrep DNA/RNA Mini Kit (Qiagen 80204) was used for gDNA extraction. The size, quality, and quantity of the purified gDNA were estimated using the 2200 Bioanalyzer (Agilent Technologies). The gDNA library was constructed using a KAPA Hyper Prep kit according to the manufacturer’s protocols (Kapa Biosystems). The quantities of the library were estimated using Qubit 3 (Thermo Fisher).

### Panel Design and Targeted Sequencing

A panel of 139 genes was used in our research (supplementary file), and the gDNA library was enriched for regions of this custom-designed probe manufactured by Agilent. The panel is designed according to the genes that have been reported to be associated with hereditary tumors based on NCCN (National Comprehensive Cancer Network) guideline, HGMD (The Human Gene Mutation Database) and TCGA (The Cancer Genome Atlas) database, as well as previously reported inherit susceptibility genes. About 750ng of library DNA was hybridized with two hybridization reagents and blocking agents of the SureSelectXT Target Enrichment System (Agilent Technologies). The enriched libraries were amplified with the P5/P7 primer. After being qualified by the 2200 Bioanalyzer, Qubit3, and a qPCR NGS library quantification kit (Agilent Technologies), the libraries were sequenced on a Hiseq X10 platform (Illumina, San Diego, CA). 18 DRGs (*ATM, ATR, BRCA1, BRCA2, BRIP1, CHEK2, FAM175A, FANCA, GEN1, MLH1, MRE11, MSH2, MSH6, NBN, PALB2, PMS2, RAD51C, RAD51D*) are grouped (1).

### Bioinformatics Analysis

Primary processing of NGS data for blood samples was performed using Trimmomatic (0.36), including demultiplexing and masking of dual-index adapter sequences. Sequences were aligned against the human reference genome (GRCh37/hg19) using BWA (version 0.7.10). IGV was used to filter alignment and sequence artifacts. Point mutations, small insertions, and deletions were identified by Samtools (version 1.3.1) and Spindel (version 0.2.5b8, 20151210). A mutation was filltered if the depth was smaller than 5, the mutational frequency was less than 20%, or was recurrently found in healthy individuals according to multiple database including NHLBI (National Heart Lung and Blood Institute) Exome Sequencing Project, 1000 Genomes Project, Exome Aggregation Consortium and gnomAD. The effects of variants were annotated using Oncotator (https://software.broadinstitute.org/cancer/cga/oncotator) and Variant Effect Predictor (https://grch37.ensembl.org/info/docs/tools/vep/index.html), as well as an in-house database (GenentronDB). The pathogenicity of germline mutations was analyzed according to the ACMG Guidelines (2015 edition), only mutations that are pathogenic/likely pathogenic and VUS (variant of uncertain significance) are investigated. All the raw data of genetic sequencing have been deposited in public GSA database (https://ngdc.cncb.ac.cn/gsa-human/, accession number: HRA001716).

### Statistical Analyses

SPSS 22.0 (IBM, USA) was used for statistical analysis. Mean age at diagnosis, TNM staging, Gleason score, and PSA level were used as classification criteria. The t-test was used for continuous variables, and the chi-square test or Fisher exact test was used for classification variables. For data that did not fit a normal distribution, nonparametric statistical tests were used. P < 0.05 was considered statistically significant.

### Pedigree Analysis

Six of the 14 patients with germline mutations in DRGs were assembled to further investigate genetic characters in their pedigrees. Four patients and their families agreed to participate in the continued study. Peripheral blood was drawn from the elbow vein of the first/second-degree relatives of these four patients; then Sanger sequencing was conducted to evaluate the hereditary feature.

## Results

### Patients

Twenty-four patients were diagnosed with PCa by prostate needle biopsy or surgical specimens. Among these patients, eight and four have first-degree and none-first-degree relatives with a history of cancer, respectively. The remaining patients have no family history of cancer. At diagnosis, the average age is 54.8 (47~62 years old), and the average PSA value is 87.5 ng/mL (minimum 5.45 ng/mL, maximum 550 ng/mL). Thirteen patients have a Gleason score of 8 or higher. At the time of diagnosis, there were 14 patients with stage T2, 6 with stage T3, and 4 with stage T4. There are six patients diagnosed with bone metastasis by isotopic bone scanning; of note 2 patients with oligometastic prostate cancer received radical resection. Four patients were diagnosed with lymph node metastasis. Nineteen patients received radical resection, and 3 received local radiotherapy. Eight patients received ADT, and 2 received ADT combined with local radiotherapy after radical resection. By MAY 1, 2021, 5 patients showed biochemical recurrence, and one developed into CRPC (**Table 1**).

**Table 1:** General information of all patients.

Category	DRGs’ mutation carriers	None carriers	P value	Total
Cases	14	10		24
Mean age of diagnosis (years old)	54	55.9	0.36 (t test)	54.8
Family history				
First degree relative	6	2		8
Non-first- degree relative	3	1		4
Gleason score at diagnosis				
≤7	5	6	0.41 (fisher exact test)	11
≥8	9	4		13
Pathological type				
IDC-P	1	0		1
TNM stage				
T1/T2	7	7	0.42 (fisher exact test)	14
T3/T4	7	3		10
Lymph node metastasis				
N1	3	1		4
Bone metastasis	4	2		6
PSA level at diagnosis				
Average value (ng/mL)	120	41.2	0.15 (ANOVA analysis)	87.5
CRPC	1	0		1

DRGs, DNA repair genes; IDC-P, intraductal carcinoma of the prostate; PSA, prostate specific antigen; CRPC, castration-resistent prostate cancer.

### Mutation Status

**Figure 1:**
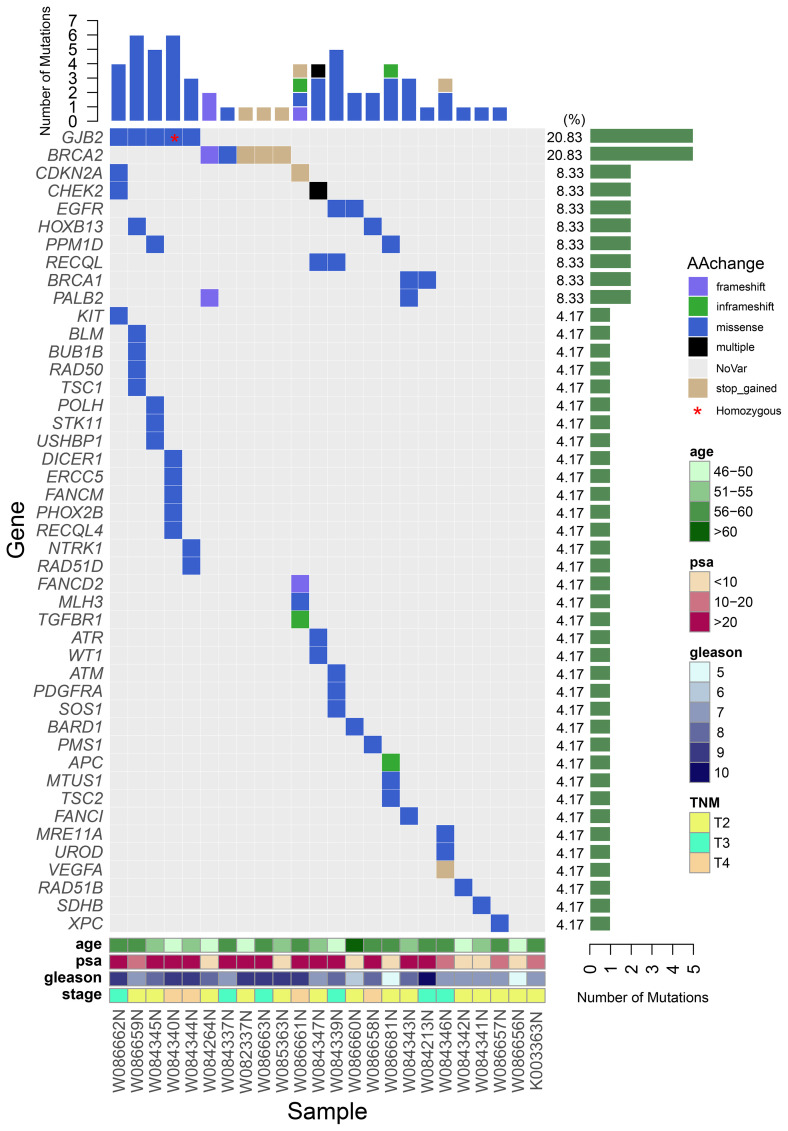
A waterfall plot shows the mutation ratio, mutation type of the mutated genes, and the clinical features of the 24 patients. The top panel shows the number of the mutations in each PCa sample; the left panel shows the frequently mutated genes; the right panel shows the detection ratio of each mutated gene; the bottom panel shows the sample number and the clinical features of the corresponding patient.

**Figure 2:**
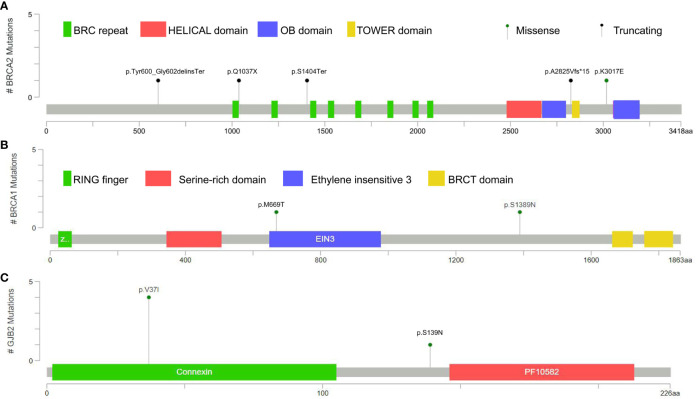
Locations of the germline mutations in BRCA2 **(A)**, BRCA1 **(B)** and GJB2 **(C)**.

A total of 62 germline mutations in 45 genes were detected in 24 peripheral venous blood samples (**Figure 1**). Missense mutation is the most common type, followed by stop-gained mutation and in-frame mutation. The detection rate of a missense mutation in all samples accounted for 82.3% (51/62). 16.1% (10/62) of the patients have either frameshift (3 frameshifts in *FANCD2, BRCA2*, and *PALB2* gene), non-frameshift (2 non-frameshift in *TGFBR1* and *APC* genes), or stop-gained mutations (5 stop-gained mutations in *BRCA2, CDKN2A*, and *VEGFA* genes), which potentially affect the functions of the encoded proteins. Interestingly, we found that fourteen patients (14/24, 58.3%) have one or more germline mutations in above-mentioned 18 DRGs. The most frequently mutant genes were *BRCA2* (5/24) and *GJB2* (5/24), followed by *CDKN2A, CHEK2, EGFR, HOXB13, PPM1D*, and *RECQL* (2/24). Mutation sites of the main genes are listed below (**Figure 2**).

### Clinical Feature

Compared to those with non-DRGs mutation carriers, the 14 patients with DRGs’ mutations have a tendency of higher overall Gleason score, higher PSA level at diagnosis, and younger ages (54 years vs 55.9 years, P=0.36). 64% (9/14 patients) of the patients with DRGs’ mutations have a Gleason score of 8 or higher compared to 40% (4/10 patients, P =0.41) of the non-carriers. The average PSA level of the DRGs mutation carriers is 120ng/mL compared to that 41.2 ng/mL (P =0.15) of the non-DRGs mutation carriers. In addition, at the time of diagnosis, 50% (7/14 patients) of patients with DRGs’ mutations have their tumor staged at T3/T4 comparing to 30% (3/10 patients, P=0.42) of the non-DRGs mutation carriers with a similar staging. Of the five patients with GJB2 mutations, two patients (one of them has homozygous GJB2 mutation) were at the T4 stage at their diagnosis with a Gleason score of 9; 1 patient at stage T3 (Gleason score of 9); 2 patients at stage T2 (Gleason score of 7 and 8, respectively). In addition, four of the five patients have a high level of PSA (>100ng/mL). Three CHEK2 mutations were detected in 2 patients at stage T2 and T3, respectively, at their diagnoses.

### Cases and Pedigrees

**Figure 3:**
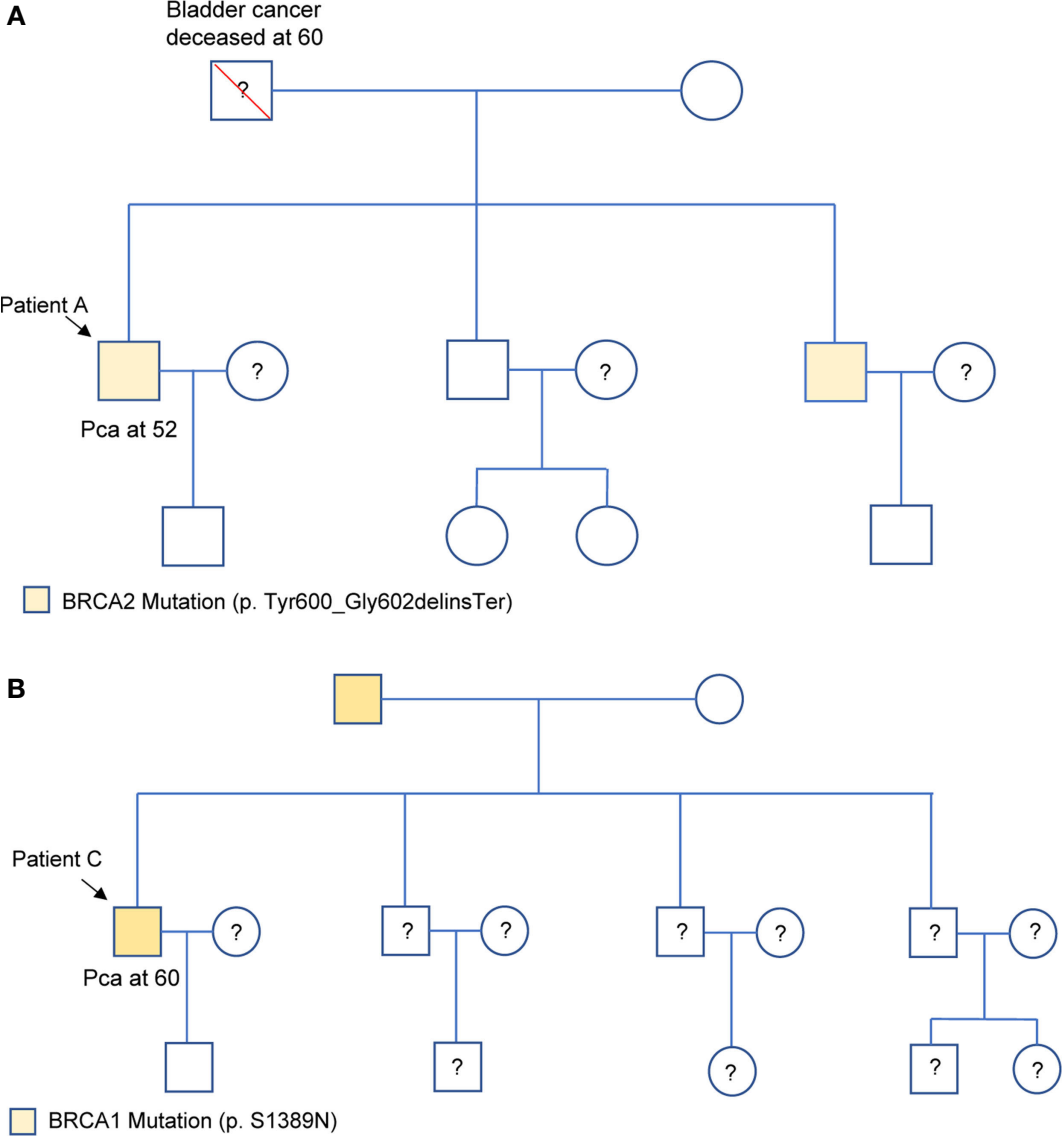
Family pedigrees of Patient A **(A)** and Patient C **(B)**, respectively. “□,○” indicate normal males and females, red “\” indicates that individual is deceased, dashes between symbols “□—○” indicate a couple, “?” indicate the mutation status is unknown, and “↘” indicate the probands. PCa represents prostate cancer.

Among the 24 patients, the data of 4 families of HRR-related mutation carriers are further analyzed. In these four pedigrees, 2 with germline mutation of *BRCA2* (Patient A and B), 1 with germline mutation of *BRCA1*, 1 with germline mutations of *BRCA2* and *PALB2* (**Table 2**). Of note, patient B with novel *BRCA2* germline mutation responded well to chemotherapy combined with ADT treatment in our previous report (2). In addition, we recently reported the other patient D with *BRCA2* and *PALB2* mutations, who responded well to radiotherapy and chemotherapy combined with ADT (3). For the other two patients, Patient A was diagnosed with PCa by needle biopsy at 52 years old, with a low PSA level of 7.95ng/dL, Gleason scores 4 + 5, imaging examination revealed no metastasis (T2bN0M0). This patient received radical resection of the prostate, and the PSA level closed to 0 during the follow-up. Results from Sanger sequencing indicate that his healthy brother shares the same germline mutation of *BRCA2* (c.1799_1804del, p.Tyr600_Gly602delinsTer), and his father died of bladder cancer at the age of 60. Patient C was diagnosed with PCa at 60 years old through needle biopsy with his PSA level of more than 550 ng/dL, Gleason scores 5 + 5, imaging examination revealed multiple bone metastasis (T3NxM1B). He received ADT combined with radiotherapy. However, his PSA reached the lowest level (0.51 ng/dL) after the radiotherapy before raising again. This patient was diagnosed as CRPC on Aug 28, 2020, then given abiraterone combined with docetaxel and nedaplatin, and PSA level slowly increased to 70 ng/dL in April of 2021. The patient and his father carry the germline mutation of *BRCA1* (c.4166G>A, p.S1389N), with no cancer history of other family members. The pedigrees of patient A and patient C are shown in **Figure 3**.

**Table 2:** Mutations status of the four patients with pedigrees.

Patient	Gene	Type of mutation	Nucleotide changes	Amino acid changes	Chromosomes	Exon	Transcript	Homozygous/heterozygous
A	*BRCA2*	Stop-gain	c.1799_1804del	p.Tyr600_Gly602delinsTer	13	10/27	NM_000059.3	heterozygous
B	*BRCA2*	Stop-gain	c.4211C>G	p.S1404Ter	13	16/27	NM_000059.3	heterozygous
C	*BRCA1*	Missense mutation	c.4166G>A	p.S1389N	17	12/22	NM_007294.3	heterozygous
D	*BRCA2*	Frameshift	c.8474_8487delCATACCCTATACAG	p.A2825Vfs*15	13	19/27	NM_000059.3	heterozygous
	*PALB2*	Frameshift	c.472delC	p.Q158Rfs*19	16	4/13	NM_024675.3	heterozygous

The symbol * means termination codon.

## Discussion

In our study, we investigated the germline mutations of 24 patients with early-onset PCa by using targeted gene sequencing of a panel of 139 genes. We found that 58.3% of the early-onset PCa patients carried DRGs’ mutations, including pathogenic/likely pathogenic and VUS. More importantly, we found that in early-onset cohorts patients with DRGs’ mutations have a tendency of higher Gleason score, higher PSA level at diagnosis, and younger ages. At last, by analyzing the case series and their pedigrees in details, we also found that patients with mutations of HRR-related genes might benefit from ADT combined with radiotherapy or platinum-based chemotherapy.

PCa is one of the most heritable cancers (4), and germline mutations in DRGs are more accountable for PCa inheritability (5), with about 22% of PCa patients harbor DRGs’ somatic mutations (6). As for germline mutations, DRGs’ mutations present approximately 4.6% in localized PCa, and 11.8% to 16.2% in metastatic disease in Caucasian population (7, 8). Whereas in Chinese population, Ye et al. have found that 9.8% PCa patients have germline mutations in their DNA repair pathway, and 6.3% of them are *BRCA2* mutations with 0.63% of either *BRCA1* or *ATM* (1). Consistently, our findings in this study show that mutations in DRGs are highly associated with PCa incidence and the early-onset of PCa. Dissimilarly, in the early-onset population of our study, we found that the frequency of PCa patients with germline mutations in DRGs was 58.3% (14/24), much higher than that in PCa patients with undefined ages (the patients diagnosed with prostate cancer at any ages, not restricted to the early-onset ages). Consistent with a recent report (18), we found that patients with DRGs’ germline mutations have higher Gleason scores, more advanced clinical stages, and more metastasis. More importantly, compared with another PCa cohort with undefined average ages in Ye et al.’s Chinese population (1), we found that early-onset PCa patients with DRGs’ mutations had a relatively younger ages (54 vs 60 years) and higher PSA levels at diagnosis (120 vs 100 ng/ml), but lower Gleason Scores (≥8, 64.3% vs 84%) and fewer metastasis (28.6% vs 71%). The discrepancy might be due to the limited sample size and that most of the mutations were VUS. Thus the results should be substantiated in a larger size of samples.

HRR pathway plays a vital role in maintaining the stability of the genome, along with multiple repair mechanisms, including base excision repair (BER) of DNA single-strand breaks, mismatch repair (MMR), and non-homologous end joining (NHEJ) (9, 10). In fact, HRR is the most efficient repair tool for DNA double-strand breaks (DSB) with high fidelity, and BRCA1/2 plays an indispensable role in this process (11, 12). Therefore, mutations in *BRCA1/2* are closely related to various cancers, especially breast and ovarian cancer in women and PCa in men (13–15). Previous study has shown that *BRCA1/2* mutations drive carcinogenesis through somatic inactivating the second wild-type allele, the “two-hit hypothesis”. Due to the combination of germline and somatic mutations, the failure of the BRCA-related HRR mechanism favour the activation of alternative and less effective DNA repair pathways, and finally result in tumor development (19, 20). Our previous report had also revealed that patient D had loss-of-heterozygosity of BRCA2 in his prostate tumors (3). In addition to DNA repair pathways, BRCA1/2 also could repress the progression of prostate cancer by inhibiting PI3K/AKT and MAP/ERK pathways, as well as MMP9 and AR signaling (21). It has been reported that PCa patients with *BRCA1/2* mutations can be treated with radiotherapy, cisplatin, anthracyclines, or poly (ADP-ribose) polymerase inhibitors (22, 23). For metastatic castration-resistant PCa with bi-allelic inactivation of *BRCA2*, chemotherapy with platinum agents has been recommended (24). In our study, the three PCa patients carrying heterozygous *BRCA2* germline mutations were treated with ADT combined with radiotherapy or chemotherapy, both primary lesions and metastases shrunk significantly, and their PSA levels were well controlled. In addition, mutations of other HRR-related factors such as ATM, ATR, BRIP1, CDK12, and CHEK2 also play vital roles in tumorigenesis and progression (16, 17). We also found that two young patients with germline mutations have relatively low PSA levels (<4ng/dL). Since the PSA level is not sensitive enough to screen PCa for these kinds of patients with DRGs’ mutations, an imaging examination should be conducted for these patients when encountering PCa-related symptoms at a young age (25).

In addition to DRGs’ mutations, mutations in *HOXB13* and *GJB2* genes have also been associated with PCa in previous studies. *HOX* is a highly conserved gene encoding homeodomain-containing transcription factors playing a vital role in body axis patterning and cell differentiation of developing embryos (26). Aberrations in *HOX* gene expression have been reported in abnormal development and malignancy, indicating that altered expression of *HOX* could be essential for both tumorigenesis and tumor suppression (27). Ewing et al. firstly found that a rare germline mutation c.252G–>A (p.G84E, rs138213197) in the first exon of *HOXB13* is associated with an increased risk of non-aggressive PCa at a young-onset (28). Soon afterward, *HOXB13* mutation (c.G216C and c.R229G) was detected in PCa patients with African and Asian ancestry as well (29). A carrier frequency of G84E mutation among European-Americans with familial young-onset PCa is 3.1%, but its frequency is threefold higher (8.4%) for both the Finnish and Swedish populations (30). Although uncommon, this mutation accounts for an eightfold increased risk of PCa diagnosed at 55 or younger (31). This mutation is more associated with hereditary PCa (OR: 6.6; 95% CI 3.3 to 12.0) (32). It has been reported that in a Finnish population that *HOXB13* G84E mutation is associated with familial young-onset (<55 years) PCa with elevated PSA (20ng/mL or higher) at the time of diagnosis. Other than the G84E mutation in *HOXB13* is mainly reported in the Western population, a similar mutation of G135E in *HOXB13* reported by LIN X et al. (33) has been associated with an increased risk of PCa in the Chinese population. However, there is no evidence showing that these mutations are associated with other clinical aggressiveness, such as higher Gleason score, progression, or recurrence. In this study, the two patients carrying the V278L germline mutation of the *HOXB13* gene were diagnosised at 50 and 55 years old, respectively. However, due to the small number of the total subjects, there is not enough relevant evidence to reveal the connection between mutations status and clinical features. Previous study also revealed that HOXB13, together with FOXA1 and GATA2, interacts with androgen receptor (AR) to promote the development and differentiation of prostate and PCa (34), while the exact mechanism of HOXB13 in carcinogenesis remains unclear.

GJB2 encodes a gap junction protein and is a putative tumor suppressor. However, more recent studies also showed that the expression level of GJB2 in metastatic tumor lesions is significantly higher than that in the primary tumor, suggesting that GJB2 may promote tumor metastasis (35). Mutations in *GJB2* gene are usually associated with non-syndromic hearing loss (NSHL) (36). It has been reported that a 17.3% biallelic mutation of the *GJB2* gene in the Chinese population (37). Since GJB2 and its isoforms express at different levels in PCa tissues, it is suggested that GJB2 may play a role in PCa tumorigenesis (38, 39). Our findings show that the rate of germline *GJB2* mutation in PCa patients is 20.8% (5/24), higher than the rate in the general populations, indicating that *GJB2* mutations might be associated with early-onset PCa. However, the role of GJB2 on PCa need to be further substantiated and the underlying mechanism remains to be unclear.

The major limitation of the present study is the small sample size, which might due to the specific particular cohort, early-onset prostate cancer. We have defined patients at ages under 65 years old as early-onset ones in our cohort according to previous studies (40–43). We noted that the current definition of early-onset prostate cancer is indeed not the classical or common used one (aged ≤55 years) according to previous report (44). However, we have a few reasons to use the current definition in our cohort. Firstly, previous study has shown that the mean age of prostate cancer patients in Asian population are higher than those in Caucasian population, suggesting a delay in prostate cancer development in Asian men (45). Secondly, the tumour latency period, the time when the tumour exists but is not detected (starts at tumour onset and ends at diagnosis by screening or clinical symptoms), might be longer in China; which might be due to the lack of routine PSA screening to certain extent. Thus most of the patients were not admitted to hospital until significant clinical symptoms appeared, evidenced by that most of the patients have advanced stages of tumors at diagnosis (46). At last, there are only a few patients with prostate cancer aged ≤55 years, who fulfilled all the inclusion criteria. Therefore, we used the age under 65 years old as early-onset in our cohort. Of note, half of the patients were diagnosed as prostate cancer at ages ≤ 55 years, only two patients were diagnosed at ages over 60 years old. Another limitation is that most of the mutations in our report are VUS. The effects of the mutations need to be further revealed.

## Conclusions

The incidence of early-onset prostate cancer increased since PSA screening was introduced. The biological differences exist between early-onset prostate cancer and late-onset disease. In our study, we found that germline mutation in DRGs is one of the major risk factors for early-onset and a more aggressive PCa. Since the PSA level in early-onset PCa with DRGs’ mutations could be relatively normal, more aggressive imaging examination is suggested than PSA screening for these kinds of patients. HRR-related mutation carriers might benefit from ADT combined with chemo/radiotherapy. Therefore, genetic testing is rather important to guide precise therapy for the patients with early-onset PCa and to direct their family members to clinical genetic counseling.

## Data Availability Statement

The datasets presented in this study can be found in online repositories. The names of the repository/repositories and accession number(s) can be found below: https://ngdc.cncb.ac.cn/gsa-human/browse/HRA001716.

## Ethics Statement

The studies involving human participants were reviewed and approved by The ethics committee of the Third Affiliated Hospital of the PLA Army Medical University (ethics number: 2018 No. 28). The patients/participants provided their written informed consent to participate in this study.

## Author Contributions

Conception/design: JJ and QL. Provision of study material or patients: TT and XT. Collection and/or assembly of data: TT, XT, ZW, YW, and JX. Data analysis and interpretation: DZ, XW, and JJ. Manuscript writing and revising: TT, XT, QL, DZ, and JJ. All authors contributed to the article and approved the submitted version.

## Funding

This study is supported by the National Natural Science Foundation of China (81802558) and the University Research Project of Army Medical University (2017XYY07, 2018XLC1014 and 2019CXLCB006).

## Conflict of Interest

Author XW was employed by Genetron Health (Beijing) Co. Changping Distric.

The authors declare that the research was conducted in the absence of any commercial or financial relationships that could be construed as a potential conflict of interest.

## Publisher’s Note

All claims expressed in this article are solely those of the authors and do not necessarily represent those of their affiliated organizations, or those of the publisher, the editors and the reviewers. Any product that may be evaluated in this article, or claim that may be made by its manufacturer, is not guaranteed or endorsed by the publisher.
