# Corrigendum: Germline mutations in patients with early-onset prostate cancer

**DOI:** 10.3389/fonc.2024.1498329

**Published:** 2024-12-18

**Authors:** Tang Tang, Xintao Tan, Ze Wang, Shuo Wang, Yapeng Wang, Jing Xu, Xiajie Wei, Dianzheng Zhang, Qiuli Liu, Jun Jiang

**Affiliations:** ^1^ Department of Urology, Daping Hospital, Army Medical University, Chongqing, China; ^2^ Genetron Health (Beijing) Co., Beijing, China; ^3^ Department of Bio-Medical Sciences, Philadelphia College of Osteopathic Medicine, Philadelphia, PA, United States

**Keywords:** prostate cancer, early-onset, next-generation sequencing, germline mutations, homologous recombination associated genes

In the published article, there was an inconsistency between **Figures 1** and **2**. The mutation types for BRCA1 and BRCA2 are not accurately represented in **Figure 1**. The information provided in **Figures 2** is correct - BRCA1 has two missense variants, and BRCA2 has 3 stop_gained variants, 1 frameshift, and 1 missense variant. The errors in **Figure 1** occurred during the figure preparation process, where the mutation types for BRCA2 in patient W082337N and BRCA1 in patient W084213N were incorrectly color-coded. The corrected **Figure 1** and its caption appear below.

**Figure 1:**
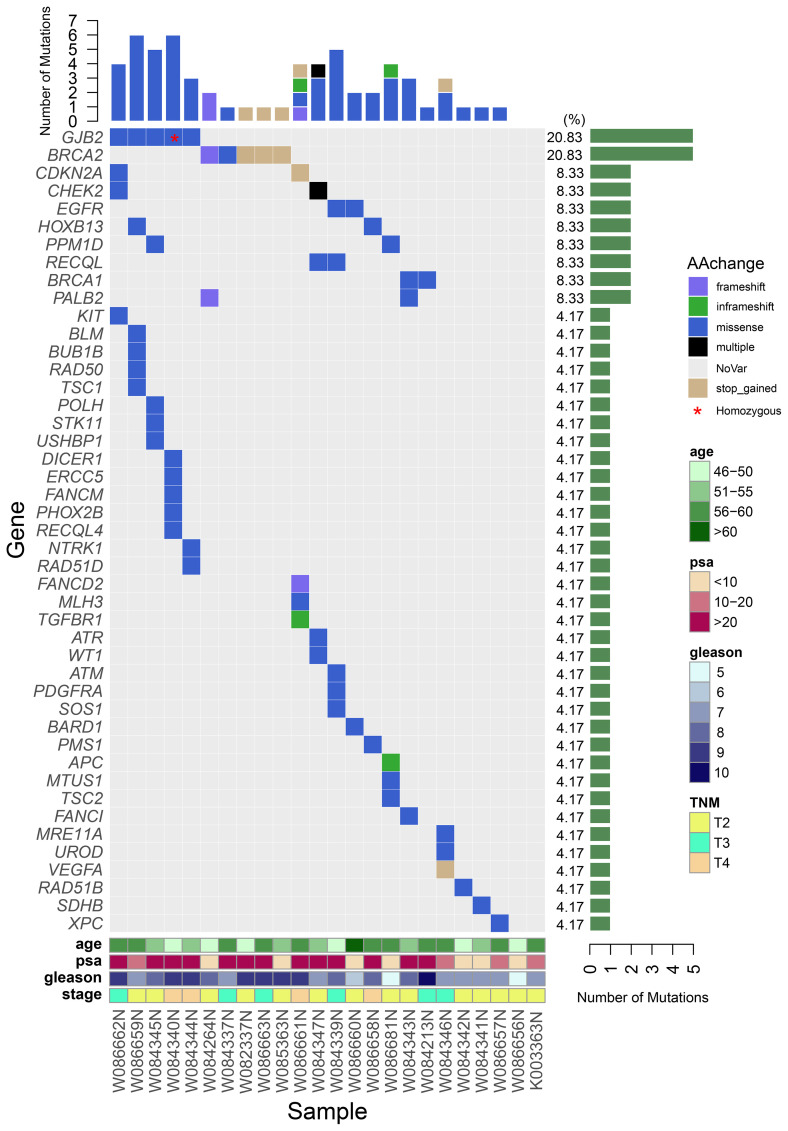
A waterfall plot shows the mutation ratio, mutation type of the mutated genes, and the clinical features of the 24 patients. The top panel shows the number of the mutations in each PCa sample; the left panel shows the frequently mutated genes; the right panel shows the detection ratio of each mutated gene; the bottom panel shows the sample number and the clinical features of the corresponding patient.

In the published article, a correction has been made to **Results**, *Cases and Pedigrees*, Paragraph 1. The mutation listed for Patient A should be BRCA2 c.1799_1804del, p.Tyr600_Gly602delinsTer, as shown in **Table 2** and **Figure 3**. This sentence previously stated:

“Results from Sanger sequencing indicate that his healthy brother shares the same germline mutation of BRCA2 (c.4211C>G, p.S1404Ter), and his father died of bladder cancer at the age of 60.”

The corrected sentence appears below:

“Results from Sanger sequencing indicate that his healthy brother shares the same germline mutation of BRCA2 (c.1799_1804del, p.Tyr600_Gly602delinsTer), and his father died of bladder cancer at the age of 60.”

The authors apologize for these errors and state that this does not change the scientific conclusions of the article in any way. The original article has been updated.

