# Supplementary material for: Germline Mutations in Patients With Early-Onset Prostate Cancer
Source: Front Oncol. 2022 Jun 6;12:826778. doi: 10.3389/fonc.2022.826778 (PMC9207501; doi:10.3389/fonc.2022.826778)
Supplement: Supplementary file 1 [file Table_1.docx]

| **Supplementary Table S1** 139 tumor susceptibility genes | | | | | | | | | | | | | | |
| --- | --- | --- | --- | --- | --- | --- | --- | --- | --- | --- | --- | --- | --- | --- |
| AIP |  | CDKN1B |  | EXT1 |  | GEN1 |  | MSH2 |  | POLH |  | SBDS |  | TP53 |
| ALK |  | CDKN1C |  | EXT2 |  | GJB2 |  | MSH6 |  | PPM1D |  | SDHA |  | TSC1 |
| APC |  | CDKN2A |  | EZH2 |  | GPC3 |  | MTAP |  | PRKAR1A |  | SDHAF2 |  | TSC2 |
| ATM |  | CEBPA |  | FANCA |  | GREM1 |  | MTUS1 |  | PRSS1 |  | SDHB |  | UROD |
| ATR |  | CHEK1 |  | FANCB |  | HMBS |  | MUTYH |  | PTCH1 |  | SDHC |  | USHBP1 |
| AXIN2 |  | CHEK2 |  | FANCC |  | HNF1A |  | NBN |  | PTCH2 |  | SDHD |  | VEGFA |
| BAP1 |  | CYLD |  | FANCD2 |  | HOXB13 |  | NF1 |  | PTEN |  | SLX4 |  | VHL |
| BARD1 |  | DDB2 |  | FANCE |  | HRAS |  | NF2 |  | PTPN11 |  | SMAD4 |  | WRN |
| BLM |  | DICER1 |  | FANCF |  | KIT |  | NSD1 |  | RAD50 |  | SMARCA4 |  | WT1 |
| BMPR1A |  | DIS3L2 |  | FANCG |  | LASP1 |  | NTRK1 |  | RAD51B |  | SMARCB1 |  | XPA |
| BRCA1 |  | EGFR |  | FANCI |  | MAX |  | PALB2 |  | RAD51C |  | SMARCE1 |  | XPC |
| BRCA2 |  | ELANE |  | FANCL |  | MC1R |  | PALLD |  | RAD51D |  | SOS1 |  | XRCC2 |
| BRIP1 |  | EPCAM |  | FANCM |  | MEN1 |  | PDGFRA |  | RB1 |  | STAT3 |  | ZMAT3 |
| BUB1B |  | ERCC1 |  | FAS |  | MET |  | PHOX2B |  | RECQL |  | STK11 |  |  |
| CBL |  | ERCC2 |  | FH |  | MITF |  | PMS1 |  | RECQL4 |  | SUFU |  |  |
| CDC73 |  | ERCC3 |  | FLCN |  | MLH1 |  | PMS2 |  | RET |  | TERT |  |  |
| CDH1 |  | ERCC4 |  | GALNT12 |  | MLH3 |  | POLD1 |  | RHBDF2 |  | TGFBR1 |  |  |
| CDK4 |  | ERCC5 |  | FATA2 |  | MRE11A |  | POLE |  | RUNX1 |  | TMEM127 |  |  |
